# Fractional-Order PID Cooperative Pose Control of Multi-Agent Rigid-Body Systems on TSE(3)*^N^*

**DOI:** 10.3390/e28101099

**Published:** 2026-10-09

**Authors:** Mohammad Maadani, Eric A. Butcher

**Affiliations:** Department of Aerospace and Mechanical Engineering, University of Arizona, 1130 N. Mountain Ave., Tucson, AZ 85721, USA; maadani@arizona.edu

**Keywords:** fractional-order control, cooperative control, multi-agent rigid body systems, pose control, Luenberger observer, Mittag–Leffler stability

## Abstract

Fractional-order PID control extends conventional PID control by allowing the integral and derivative orders to take non-integer values, thereby introducing α and β as additional tuning parameters beyond the conventional PID gains. Two strategies for fractional-order ${\mathrm{PI}}^{\beta }D^{\alpha }$ cooperative pose control of a multi-agent rigid body system are considered assuming both undirected and directed communication networks, together with Caputo fractional derivative and integral operators. The rigid-body pose configurations are described using exponential coordinates associated with the Lie group SE(3), while the state space is $\mathrm{TSE}{\left(3\right)}^{N}$. Analytical stability bounds are obtained using Matignon’s theorem in terms of the proportional, derivative, and integral gains for the attitude and translational controllers, as well as the fractional orders, that guarantee global Mittag–Leffler stability. The fractional control strategy provides additional flexibility in tuning the closed-loop response beyond that of conventional integer-order PID control. Since the fractional derivative states are not directly measurable, a Luenberger observer is employed to estimate these states from the measurable pose exponential coordinates, and the resulting estimates are used in the implementation of the feedback protocols. Simulation examples of formation establishment further incorporate cohesion and collision-avoidance strategies based on artificial potential functions, illustrating the effectiveness and practical implementation of the proposed fractional ${\mathrm{PI}}^{\beta }D^{\alpha }$ control protocols.

## 1. Introduction

Cooperative control of networked rigid-body systems has received significant attention over the past few decades due to its broad range of applications. In many practical cooperative control problems, the coupling between attitude and translational motion requires the simultaneous consideration of the full six-degree-of-freedom (6-DOF) pose of each agent. Treating rotational and translational dynamics within a unified framework leads to a more compact and efficient formulation for the analysis and control of rigid-body motion [1]. To avoid the singularities, non-uniqueness, and discontinuous control associated with attitude parameterizations, control algorithms can be formulated directly on the Lie groups SO(3) and SE(3), or equivalently on their exponential coordinates, where the rotational component is represented by the principal rotation vector. This geometric framework naturally captures the coupled rotational and translational dynamics of rigid bodies [2].

Several studies have addressed cooperative control of multi-agent rigid-body systems using proportional–integral–derivative (PID)-type feedback. The 6-DOF consensus problem was first investigated in [3] using a proportional–derivative (PD) controller formulated in exponential coordinates. Prescribed-time pose consensus, in which the agents are guaranteed to reach pose consensus within a user-specified time, was later developed in [4] using exponential coordinates. In [5], two consensus algorithms were proposed for multi-agent spacecraft in orbital relative motion, one of which employed a PID consensus protocol based on the exponential coordinates of SE(3). The integer-order PID consensus problem for general multi-agent rigid-body systems was subsequently studied in [6]. Related PID-based synchronization and formation control approaches have also been proposed for rigid-body attitude synchronization [7] and formation control of networked vehicles under constant and time-varying disturbances [8]. More recently, a general high-order linear consensus framework for directed communication graphs with partial relative-state information and a reference model was developed in [9].

Recent research has also focused on cooperative control of fractional-order multi-agent systems, where integer-order feedback controllers are applied to fractional-order agent dynamics. For example, Chen et al. [10] investigate robust consensus of fractional-order multi-agent systems with input saturation and external disturbances under directed communication topologies. Gong [11] developed adaptive leaderless and leader-follower consensus algorithms for nonlinear fractional-order multi-agent systems, while Liu et al. [12] proposed a formation control strategy that accounts for relative damping and nonuniform communication delays.

A conventional PID controller combines proportional, integral, and derivative feedback to shape the transient and steady-state response of a dynamical system. Fractional-order PID control generalizes this structure by allowing the orders of the integral and derivative terms to take non-integer values. Thus, a ${\mathrm{PI}}^{\beta }D^{\alpha }$ controller introduces the fractional integral order β and fractional derivative order α as additional design parameters beyond the conventional PID gains. Fractional-order control laws, which incorporate fractional derivatives and integrals in the feedback loop, offer additional flexibility for shaping the closed-loop response and improving stability by allowing the derivative and integral orders to be tuned independently [13]. For a single rigid body, Nazari et al. [14] proposed fractional-order attitude control laws based on both SO(3) and the principal rotation vector. Nevertheless, fractional-order PID cooperative control has received comparatively little attention. Yaylali et al. [15] developed a fractional-order PID consensus controller for a double-integrator multi-agent system, where the derivative feedback was allowed to have a non-integer order. Stability was established using Matignon’s theorem for linear time-invariant fractional-order systems; however, only undirected communication topologies were considered, and analytical stability conditions were derived only for complete graphs. More recently, Maadani and Butcher [16] extended this framework to fractional-order attitude and pose consensus of multi-agent rigid-body systems using exponential coordinates under undirected communication graphs. Although Matignon’s theorem was employed to analyze stability for several representative cases, analytical necessary and sufficient stability conditions explicitly relating the proportional, derivative, and integral gains to the fractional derivative and integral orders were not established.

In this paper, the fractional-order PID cooperative pose control problem for multi-agent rigid-body systems is studied using exponential coordinates together with Caputo fractional derivatives and fractional integrals. This work extends the results of [14,17] to cooperative control of networked rigid-body systems. Unlike Maadani and Butcher [16], the present work considers both undirected and directed communication topologies and derives analytical necessary and sufficient stability conditions using Matignon’s theorem. Two fractional-order PID cooperative control strategies are proposed: one based on feedback of relative pose states together with their fractional derivatives and integrals, and another based on feedback of fractional-order state errors relative to a reference model. Since the fractional derivative states are not directly measurable, a Luenberger observer is introduced to estimate these states from the measurable pose exponential coordinates, enabling practical implementation of the proposed controllers. Hence, unlike Butcher and Maadani [18], who assume that the fractional derivative states are available for feedback, the proposed approach relies only on measurable pose states and uses an observer to estimate the fractional states required for feedback. The main contribution is the derivation of analytical stability bounds involving the separate proportional, derivative, and integral gains for both the attitude and translational dynamics together with the fractional derivative and integral orders, thereby guaranteeing global Mittag–Leffler stability of the closed-loop system. Finally, simulation examples involving formation establishment together with cohesion and collision-avoidance strategies based on artificial potential functions demonstrate the effectiveness of the proposed controllers and validate the derived stability conditions. In the pose-consensus setting considered here, the fractional derivative and integral orders provide additional tuning parameters beyond the proportional, derivative, and integral gains of a conventional integer-order PID controller. This additional flexibility can be used to modify the transient convergence characteristics of the networked rigid-body system. As demonstrated in the numerical results, the fractional-order controller achieves a shorter settling time than its integer-order counterpart under the same simulation conditions.

## 2. Preliminaries

### 2.1. Rigid Body Dynamics

The attitude of a rigid body is expressed by the rotation matrix *R* from the body-fixed coordinate frame B to the inertial frame N, which is an element of the real special orthogonal group $\mathrm{SO}\left(3\right)\;=\{R\in R^{3\times 3}|\;R^{T}R=I_{3}$, det(R)=1}, the 3-dimensional Lie group matrix representation of rigid body attitude. The 6-dimensional configuration (accounting for translation and orientation) space of rigid body pose is the (non-Abelian) Lie group SE(3), a nonlinear manifold which can be expressed as the semi-direct product $\mathrm{SE}\left(3\right)=R^{3}⋉\mathrm{SO}\left(3\right)$, where $R^{3}$ is the configuration space of the position of the center of mass. An element of SE(3) has the matrix representation given by the 4×4 matrix

(1) $$\begin{array}{c} g=\left[\begin{array}{cc} R & r\\ 0_{1\times 3} & 1 \end{array}\right]\in \mathrm{SE}\left(3\right), \end{array}$$

where R∈SO(3) and $r\in R^{3}$ is the position vector from the origin of the inertial frame to the center of mass of the rigid body expressed in the inertial frame. The Lie algebra of SE(3), denoted by se(3), is a six-dimensional vector space that is tangent to SE(3) at the identity element. Similarly, so(3) is the Lie algebra corresponding to SO(3). The pose of the rigid body can be alternatively expressed with the exponential coordinates $X={\left[\Theta^{T},p^{T}\right]}^{T}\in R^{6}$ using the logarithm map

(2) $$\begin{array}{c} X^{\wedge }=\left[\begin{array}{cc} \Theta_{3\times 3}^{\times } & p_{3\times 1}\\ 0_{1\times 3} & 0 \end{array}\right]=\log\left(g\right)\in \mathrm{se}\left(3\right) \end{array}$$

in which $\Theta^{\times }=\log\left(R\right)\in \mathrm{so}\left(3\right)$ and $p\in R^{3}$ are the exponential coordinates for attitude (principal rotation vector or PRV) and position, respectively. The set of PRVs is the closed unit ball of radius π in $R^{3}$. Every PRV inside the ball uniquely corresponds to a certain physical attitude, while there are two PRVs on the surface of the ball corresponding to a given attitude (rotation by π about some axis).

The augmented velocity vector of the rigid body is

(3) $$\begin{array}{c} \Omega ={\left[\omega^{T},v^{T}\right]}^{T}\in R^{6}, \end{array}$$

where $\omega \in R^{3}$ and $v\in R^{3}$ denote the angular and translational velocities, respectively, expressed in the body frame. The attitude kinematics of a rigid body are described in terms of the PRV Θ by

(4) $$\begin{array}{c} \dot{\Theta }=A\left(\Theta \right)\omega \end{array}$$

where

(5) $$\begin{array}{c} A\left(\Theta \right)=I_{3}+\frac{1}{2}\Theta^{\times }+\left(\frac{1}{\theta^{2}}-\frac{1+\cos\theta }{2\theta \sin\theta }\right){\left(\Theta^{\times }\right)}^{2} \end{array}$$

and θ=∥Θ∥ where 0≤θ≤π is the corresponding principal rotation angle (PRA). The closed-form expression of the inverse matrix of A(Θ) is denoted by

(6) $$\begin{array}{c} S\left(\Theta \right)=A^{-1}\left(\Theta \right)=I_{3}-\left(\frac{1-\cos\theta }{\theta^{2}}\right)\Theta^{\times }+\left(\frac{\theta -\sin\theta }{\theta^{3}}\right){\left[\Theta^{\times }\right]}^{2} \end{array}$$

Similarly, the kinematics of the exponential coordinates associated with SE(3) are given by

(7) $$\begin{array}{c} \dot{X}=B\left(X\right)\Omega , \end{array}$$

where the operator B(·) is a 6×6 matrix expressed as

(8) $$\begin{array}{c} B\left(X\right)=I_{6}+\frac{1}{2}{\mathrm{ad}}_{X}+a\left(\theta \right){\mathrm{ad}}_{X}^{2}+b\left(\theta \right){\mathrm{ad}}_{X}^{4}=\left[\begin{array}{cc} A(\Theta ) & 0_{3\times 3}\\ T(\Theta ,p) & A(\Theta ) \end{array}\right] \end{array}$$

where a(·) and b(·) are nonlinear functions in terms of the principal rotation angle given by

$$\begin{array}{c} a\left(\theta \right)=\frac{1}{\theta^{2}}\left(2-\frac{3}{4}\theta \cot\left(\frac{\theta }{2}\right)-\frac{\theta^{2}}{8}\csc^{2}\left(\frac{\theta }{2}\right)\right)\\ b\left(\theta \right)=\frac{1}{\theta^{4}}\left(1-\frac{1}{4}\theta \cot\left(\frac{\theta }{2}\right)-\frac{\theta^{2}}{8}\csc^{2}\left(\frac{\theta }{2}\right)\right) \end{array}$$

and the adjoint operator ${\mathrm{ad}}_{\Omega }$ is defined as

(9) $$\begin{array}{c} {\mathrm{ad}}_{\Omega }=\left[\begin{array}{cc} \omega^{\times } & 0_{3\times 3}\\ v^{\times } & \omega^{\times } \end{array}\right]\in R^{6\times 6} \end{array}$$

In (8) the matrix T(Θ,p) is defined as

(10) $$\begin{array}{cc} T(\Theta ,p) & =\frac{1}{2}{\left(S{\left(\Theta \right)}^{T}p\right)}^{\times }A\left(\Theta \right)+\left(\frac{1}{\theta^{2}}-\frac{1+\cos\theta }{2\theta \sin\theta }\right)\left(\Theta p^{T}+\left(\Theta^{T}p\right)A\left(\Theta \right)\right)\\ \; & -\frac{\left(1+\cos\theta )(\theta -\sin\theta \right)}{2\theta \sin^{2}\theta }S{\left(\Theta \right)}^{T}p\Theta^{T}+\left(\frac{\left(1+\cos\theta )(\theta +\sin\theta \right)}{2\theta^{3}\sin^{2}\theta }-\frac{2}{\theta^{4}}\right)\Theta^{T}p\Theta \Theta^{T} \end{array}$$

Hence, the closed-form expression of the $B^{-1}\left(X\right)$ matrix can be represented as [1]

(11) $$B^{-1}\left(X\right)=\left[\begin{array}{cc} A^{-1}\left(\Theta \right) & 0_{3\times 3}\\ -A^{-1}\left(\Theta \right)T\left(\Theta ,p\right)A^{-1}\left(\Theta \right) & A^{-1}\left(\Theta \right) \end{array}\right]$$

Also, the kinetics of a rigid body pose can be expressed in its body frame as

(12) $$\begin{array}{c} \dot{\Omega }=I^{-1}\left({\mathrm{ad}}_{\Omega }^{*}I\Omega +u\right) \end{array}$$

where $u={\left[\tau^{T},\phi^{T}\right]}^{T}\in R^{6}$ is the control input, $\tau \in R^{3}$ and $\phi \in R^{3}$ denote the control torque and force inputs, respectively, in the body frame. The co-adjoint operator is defined as

(13) $$\begin{array}{c} {\mathrm{ad}}_{\Omega }^{*}={\mathrm{ad}}_{\Omega }^{T}=\left[\begin{array}{cc} -\omega^{\times } & -v^{\times }\\ 0_{3\times 3} & -\omega^{\times } \end{array}\right] \end{array}$$

and the 6×6 matrix of mass ($m\in R^{+}$) and inertia ($J\in D\subset R^{3\times 3}$) properties is given by

(14) $$\begin{array}{c} I=\left[\begin{array}{cc} J & 0_{3\times 3}\\ 0_{3\times 3} & mI_{3} \end{array}\right] \end{array}$$

### 2.2. Communication Graph

Suppose there are *N* agents in a network defined by the communication topology G(V,E,A), where $V=\{v_{1},\ldots ,v_{N}\}$ is the set of vertices (agents), E⊆V×V is the set of directed edges, and $A=[w_{ij}]$ is the nonnegative weighted adjacency matrix. Here, $w_{ij}>0$ if and only if (j,i)∈E, indicating that information flows from agent *j* to agent *i*. Accordingly, the set of neighbors of agent *i* is defined as $N_{i}=\left\{j\in V:\left(j,i\right)\in E\right\},$ i.e., the set of agents from which agent *i* can directly receive information. The vertex strength matrix of the graph G is a diagonal matrix D, where the diagonal element $d_{ii}$ is given by

(15) $$\begin{array}{c} d_{ii}=\sum_{\begin{array}{c} k=1\\ k\neq i \end{array}}^{N}w_{ik},\;i=1,\ldots ,N. \end{array}$$

Accordingly, the graph Laplacian matrix is defined as L=D−A. Let $\mu_{j}=a_{j}\pm ib_{j}$ be the *j*th eigenvalue of L. For an undirected graph the eigenvalues are real and non-negative.

Lemma 2.4 in [19] states that L has a simple zero eigenvalue $\mu_{1}=0$, with corresponding right and left eigenvectors $1={\left(1,\ldots ,1\right)}^{T}$ and *p*, respectively, and that all remaining eigenvalues have positive real parts if and only if G(V,E,A) contains a spanning tree. For a connected undirected graph, the left eigenvector may be chosen as p=1.

### 2.3. Riemann–Liouville Fractional Integral

The Riemann–Liouville fractional integral is a commonly used generalization of the integer-order integral to arbitrary positive orders. For a function f(t), the Riemann–Liouville fractional integral of order β>0 is defined as

(16) Itβ0f(t)=1Γ(β)∫0t(t−τ)β−1f(τ)dτ,

where Γ(·) denotes the Gamma function. The subscript 0 indicates that the lower terminal of the fractional integral is t=0. For integer values of β, the Riemann–Liouville fractional integral reduces to the corresponding repeated integer-order integral. In particular, for β=1,

(17) It10f(t)=∫0tf(τ)dτ.

In what follows, the simplified notation

(18) Itβ0f(t)≡D−βf(t)

is adopted for the fractional integral of order β.

### 2.4. Caputo Fractional Derivative

The Caputo derivative [20] is a commonly used definition of a fractional derivative. The Caputo fractional derivative of order α of a function f(t) is defined as

(19) Dtα0Cf(t)=1Γ(⌈α⌉−α)∫0tf(⌈α⌉)(τ)(t−τ)α+1−⌈α⌉dτ,

where ⌈.⌉ is the ceiling function. In contrast to the other definitions of the fractional derivative, the Caputo derivative is particularly useful in physical and engineering applications because it allows for the use of standard initial conditions when used in fractional differential equations. In what follows, it is always assumed that the Caputo derivative is being used, with the simplified notation Dtα0C→Dα.

### 2.5. Matignon’s Theorem

For integer-order systems, it is well known that an LTI system of the form $\dot{x}=Mx$ is asymptotically stable if the matrix *M* is Hurwitz, or equivalently, if all eigenvalues of *M* are in the left-half complex plane. Similarly, the fractional pseudo-state differential equation $D^{\alpha }x=Mx$ of dimension *n* is stable if the eigenvalues $\lambda_{i}$ of *M* satisfy

(20) $$\begin{array}{c} |\mathrm{Arg}\left(\lambda_{i}\right)|>\frac{\alpha \pi }{2},\;\forall i=1,2,\ldots ,n. \end{array}$$

**Figure 1:**
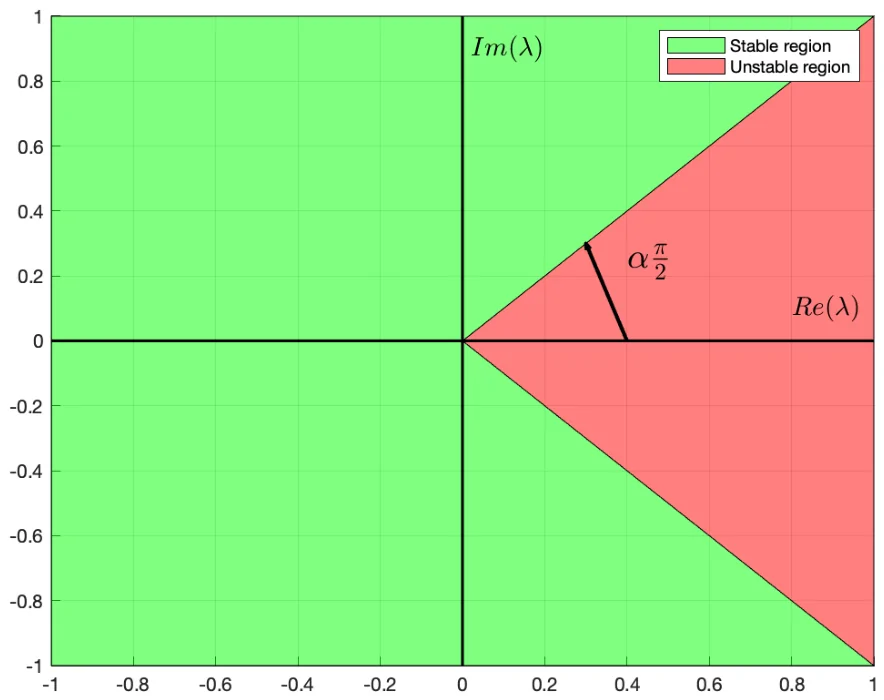
Stable and unstable regions according to Matignon’s criterion.

This is known as Matignon’s theorem [21], and Figure 1 shows the stable and unstable regions. Note that the condition in (20) reduces to the Hurwitz condition for integer-order systems when α→1. Linear fractional order systems that satisfy Matignon’s theorem are stable in the Mittag–Leffler sense [13].

### 2.6. Luenberger-Type Fractional-Order Linear Observer

State estimation is a fundamental problem in control theory. For linear systems, a classical solution is provided by the well-known Luenberger observer, which reconstructs unmeasured states using available output measurements and knowledge of the system dynamics. This concept can be extended to fractional-order systems through the design of a fractional-order observer [22].

Consider the fractional-order linear system

(21) $$\begin{array}{cc} D^{\alpha }x\left(t\right) & =Ax(t)+Bu(t), \end{array}$$

(22) $$\begin{array}{cc} y(t) & =Cx(t), \end{array}$$

where α denotes the fractional order, $x\in R^{n}$ is the state vector, $u\in R^{m}$ is the control input, and $y\in R^{p}$ is the measured output. A Luenberger-type fractional-order observer is defined as

(23) $$\begin{array}{c} D^{\alpha }\hat{x}\left(t\right)=A\hat{x}\left(t\right)+Bu\left(t\right)+L\;\left(y\left(t\right)-C\hat{x}\left(t\right)\right), \end{array}$$

where $\hat{x}\left(t\right)\in R^{n}$ denotes the estimated state vector and $L\in R^{n\times p}$ is the observer gain matrix. Defining the estimation error as

(24) $$\begin{array}{c} e\left(t\right)=x\left(t\right)-\hat{x}\left(t\right), \end{array}$$

and subtracting (23) from (21), the observer error dynamics are obtained as

(25) $$\begin{array}{c} D^{\alpha }e\left(t\right)=\left(A-LC\right)e\left(t\right). \end{array}$$

According to Matignon’s stability criterion [23], the observer estimation error converges asymptotically to zero if all eigenvalues of the matrix (A−LC) satisfy

(26) $$\begin{array}{c} \left|\arg\;\left(\lambda_{i}\left(A-LC\right)\right)\right|>\frac{\alpha \pi }{2},\;i=1,\ldots ,n. \end{array}$$

Therefore, the observer gain matrix *L* must be designed such that all eigenvalues of the observer error matrix (A−LC) lie within the stability region of the fractional-order system.

## 3. Fractional Cooperative Control Protocols

### 3.1. Agent and Error Dynamics

Assume there are *N* rigid body agents with inertia matrices $J_{i}$ and masses $m_{i}$, i=1,…,N, which share states according to a communication graph G(V,E,A). The 6-DOF dynamics of the *i*th agent are given by

(27) $$\begin{array}{cc} {\dot{X}}_{i} & =B\left(X_{i}\right)\Omega_{i}, \end{array}$$

(28) $$\begin{array}{cc} I_{i}{\dot{\Omega }}_{i} & ={\mathrm{ad}}_{\Omega_{i}}^{*}I_{i}\Omega_{i}+u_{i} \end{array}$$

where $X_{i}$ and $\Omega_{i}$ are the exponential coordinates and the augmented velocity vector of the *i*th agent, respectively. From (27), it is obtained that

(29) $$\begin{array}{c} \Omega_{i}=U\left(X_{i}\right){\dot{X}}_{i},\;i=1,\ldots ,N \end{array}$$

where $U\left(X_{i}\right)=B^{-1}\left(X_{i}\right)$. By taking the time derivative of (29) and multiplying both sides by $I_{i}$, it is seen that

(30) $$\begin{array}{c} I_{i}{\dot{\Omega }}_{i}=I_{i}\dot{U}\left(X_{i},{\dot{X}}_{i}\right){\dot{X}}_{i}+I_{i}U\left(X_{i}\right){\ddot{X}}_{i} \end{array}$$

where the derivation of $\dot{U}\left(X_{i},{\dot{X}}_{i}\right)$ is provided in the Appendix A. Combining (28) and (30) yields

(31) $$\begin{array}{c} I_{i}\dot{U}\left(X_{i},{\dot{X}}_{i}\right){\dot{X}}_{i}+I_{i}U\left(X_{i}\right){\ddot{X}}_{i}-{\mathrm{ad}}_{\Omega_{i}}^{*}I_{i}U\left(X_{i}\right){\dot{X}}_{i}=u_{i} \end{array}$$

Using the change in variables

(32) $$\begin{array}{cc} G(X_{i},I_{i}) & =I_{i}U\left(X_{i}\right), \end{array}$$

(33) $$\begin{array}{cc} H(X_{i},\Omega_{i},I_{i}) & =I_{i}\dot{U}\left(X_{i},{\dot{X}}_{i}\right)-{\mathrm{ad}}_{\Omega_{i}}^{*}I_{i}U\left(X_{i}\right), \end{array}$$

(31) can be written as

(34) $$\begin{array}{c} G\left(X_{i},I_{i}\right){\ddot{X}}_{i}+H\left(X_{i},\Omega_{i},I_{i}\right){\dot{X}}_{i}=u_{i} \end{array}$$

Define the error of the *i*th agent’s state as ${\tilde{X}}_{i}=X_{i}-X_{i}^{d}$ where $X_{i}^{d}=\left[0_{1\times 3},{\left(p_{i}^{d}\right)}^{T}\right]$ and $p_{i}^{d}$ is the constant desired exponential position coordinate of agent *i* defined from its center of formation. Furthermore, define $X_{ij}^{d}={\left[0_{1\times 3},\;{p_{ij}^{d}}^{T}\right]}^{T}=X_{i}^{d}-X_{j}^{d}$, while $p_{ij}^{d}$ is the chosen constant desired separation between rigid bodies *i* and *j* for the formation such that ${\tilde{X}}_{i}-{\tilde{X}}_{j}=X_{i}-X_{j}-X_{ij}^{d}$. Then, the open loop error dynamics of (34) can be written as

(35) $$\begin{array}{c} G\left(X_{i},I_{i}\right){\ddot{\tilde{X}}}_{i}+H\left(X_{i},\Omega_{i},I_{i}\right){\dot{\tilde{X}}}_{i}=u_{i} \end{array}$$

### 3.2. Feedback of Relative States Only with an Undirected Graph

The first fractional cooperative control protocol $u_{i}$ is designed as

(36) $$\begin{array}{cc} u_{i} & =H\left(X_{i},\Omega_{i},I_{i}\right){\dot{X}}_{i}\\ & -G\left(X_{i},I_{i}\right)\sum_{j∼i}\left(K_{I}D^{-\beta }\left(X_{i}-X_{j}-X_{ij}^{d}\right)+K_{P}\left(X_{i}-X_{j}-X_{ij}^{d}\right)+K_{D}D^{\alpha }\left(X_{i}-X_{j}\right)\right)\\ \; & =H\left(X_{i},\Omega_{i},I_{i}\right){\dot{\tilde{X}}}_{i}-G\left(X_{i},I_{i}\right)\sum_{j∼i}\left(K_{I}D^{-\beta }\left({\tilde{X}}_{i}-{\tilde{X}}_{j}\right)+K_{P}\left({\tilde{X}}_{i}-{\tilde{X}}_{j}\right)+K_{D}D^{\alpha }\left({\tilde{X}}_{i}-{\tilde{X}}_{j}\right)\right) \end{array}$$

where α∈(0,2) and β∈(0,1] denote the fractional derivative and fractional integral orders, respectively. Also, $K_{I}$, $K_{P}$, and $K_{D}$ are the integral, proportional, and derivative control gain matrices, respectively, defined as

$$\begin{array}{c} K_{I}=\left[\begin{array}{cc} k_{I_{A}}I_{3} & 0_{3}\\ 0_{3} & k_{I_{T}}I_{3} \end{array}\right],\;K_{P}=\left[\begin{array}{cc} k_{P_{A}}I_{3} & 0_{3}\\ 0_{3} & k_{P_{T}}I_{3} \end{array}\right],\;K_{D}=\left[\begin{array}{cc} k_{D_{A}}I_{3} & 0_{3}\\ 0_{3} & k_{D_{T}}I_{3} \end{array}\right] \end{array}$$

while $k_{P_{A}}$, $k_{I_{A}}$, and $k_{D_{A}}$ are the attitude control scalar gains, and $k_{P_{T}}$, $k_{I_{T}}$, and $k_{D_{T}}$ are the translational control scalar gains.

The control law in (36) is presented in its ideal full-state feedback form and is used for the subsequent stability analysis. In practice, however, the relative exponential-coordinate errors are assumed to be directly available from measurements and are therefore used in the proportional and integral feedback terms. Since the generalized velocity (equivalently, the fractional derivative state) is not directly measurable, the derivative feedback term is implemented using the observer estimate. Consequently, the observer is employed only to reconstruct the derivative-related states required for implementation, while the nominal closed-loop dynamics remain those derived below.

By combining (35) and (36) and using ${\tilde{X}}^{T}=\left({\tilde{X}}_{1}^{T},\ldots ,{\tilde{X}}_{N}^{T}\right)$, the closed-loop system can be written as

(37) $$\begin{array}{c} {\ddot{\tilde{X}}}_{i}=-K_{I}D^{-\beta }\sum_{j∼i}\left({\tilde{X}}_{i}-{\tilde{X}}_{j}\right)-K_{P}\sum_{j∼i}\left({\tilde{X}}_{i}-{\tilde{X}}_{j}\right)-K_{D}D^{\alpha }\sum_{j∼i}\left({\tilde{X}}_{i}-{\tilde{X}}_{j}\right) \end{array}$$

for i=1,…,N. Defining the stacked vector $\tilde{X}={\left[{\tilde{X}}_{1}^{T},\ldots ,{\tilde{X}}_{N}^{T}\right]}^{T}$, the closed-loop system can be expressed compactly as

(38) $$\begin{array}{c} \ddot{\tilde{X}}=-\left(L⊗K_{I}\right)D^{-\beta }\tilde{X}-\left(L⊗K_{P}\right)\tilde{X}-\left(L⊗K_{D}\right)D^{\alpha }\tilde{X}. \end{array}$$

Suppose the fractional orders are rational. Let $\alpha =\frac{p_{\alpha }}{q_{\alpha }}$ and $\beta =\frac{p_{\beta }}{q_{\beta }}$, and let *r* denote the least common multiple (LCM) of $q_{\alpha }$ and $q_{\beta }$, i.e., $r=m_{\alpha }q_{\alpha }=m_{\beta }q_{\beta }$, where $m_{\alpha }$, $m_{\beta }$, $p_{\alpha }$, $q_{\alpha }$, $p_{\beta }$, and $q_{\beta }$ are positive integers. Define an auxiliary state vector as $z_{i}^{T}=\left[\xi_{i1}^{T},\xi_{i2}^{T},\ldots ,\xi_{in}^{T}\right]$ for the *i*-th agent, where $n=m_{\beta }\left(p_{\beta }+2q_{\beta }\right)=m_{\beta }p_{\beta }+2r$, $\xi_{i1}=D^{-\beta }{\tilde{X}}_{i}\in R^{6}$, and $\xi_{ij}=D^{\zeta }\xi_{i(j-1)}\in R^{6}$ for j=2,…,n with $\zeta =\frac{1}{r}$. With these definitions and using $Z^{T}=\left(z_{1}^{T},\ldots ,z_{N}^{T}\right)$, the local closed-loop system of (38) is

(39) $$\begin{array}{c} D^{\zeta }Z=\left(\left(I_{N}⊗A⊗I_{6}\right)-\left(L⊗BK_{A}⊗\left[\begin{array}{cc} I_{3} & 0_{3}\\ 0_{3} & 0_{3} \end{array}\right]\right)-\left(L⊗BK_{T}⊗\left[\begin{array}{cc} 0_{3} & 0_{3}\\ 0_{3} & I_{3} \end{array}\right]\right)\right)Z=WZ \end{array}$$

where

$$\begin{array}{cc} A & ={\left[\begin{array}{ccccc} 0 & 1 & \ldots & 0 & 0\\ 0 & 0 & 1 & \ldots & 0\\ \vdots & 0 & \ddots & 1 & 0\\ 0 & 0 & \ldots & 0 & 1\\ 0 & 0 & \ldots & 0 & 0 \end{array}\right]}_{n\times n}\\ B^{T} & ={\left[0,0,\ldots ,0,1\right]}_{1\times n}\\ K_{A} & ={\left[k_{I_{A}},\ldots ,k_{P_{A}},\ldots ,k_{D_{A}},\ldots \right]}_{1\times n},\\ K_{T} & ={\left[k_{I_{T}},\ldots ,k_{P_{T}},\ldots ,k_{D_{T}},\ldots \right]}_{1\times n} \end{array}$$

While $k_{I_{A}}$ and $k_{I_{T}}$ occupy the first entries of $K_{A}$ and $K_{T}$, respectively, the locations of $k_{P_{A}}$, $k_{D_{A}}$, $k_{P_{T}}$, and $k_{D_{T}}$ are determined by the fractional orders, whereas all remaining matrix entries are zero. Since L has a zero eigenvalue, *W* has 6n zero eigenvalues.

Using the Matignon’s theorem (20) and evaluating the eigenvalues $\lambda_{i},i=1,\ldots ,6Nn$ of *W* in (39), it can be shown that the closed-loop system is stable and each pair of agents achieves a desired relative configuration (i.e., attitude synchronization while establishing the desired formation) asymptotically, as long as the non-zero eigenvalues of *W* satisfy $|\mathrm{Arg}(\lambda_{i})|>\pi \zeta /2$. Note that, for the case of integer order PID control where α=β=1, the matrices above are given by

$$\begin{array}{c} A=\left[\begin{array}{ccc} 0 & 1 & 0\\ 0 & 0 & 1\\ 0 & 0 & 0 \end{array}\right],\;B^{T}=\left[0,0,1\right],\;K_{A}=\left[k_{I_{A}},k_{P_{A}},k_{D_{A}}\right],\;K_{T}=\left[k_{I_{T}},k_{P_{T}},k_{D_{T}}\right]. \end{array}$$

while Matignon’s theorem reduces to the left half complex plane.

In what follows, the stability bounds for the control gains in closed-loop system (37) are presented.

**Theorem** **1.** *Assume that N bodies communicate via an undirected communication topology G(V,E,A) with real Laplacian eigenvalues $\mu_{j}$. Also, suppose that $k_{I}$, $k_{P}$, and $k_{D}$ represent the gains for either the attitude or translational dynamics. Employing the fractional ${\mathrm{PI}}^{\beta }D^{\alpha }$ consensus protocol (36), the closed-loop system (37) has Mittag–Leffler consensus stability if G(V,E,A) is connected, $k_{I},k_{D}>0$ and $k_{P}>k_{P}^{★}$, where*

(40) $$\begin{array}{cc} k_{P}^{★}=\frac{1}{\mu_{2}}{\left(\frac{k_{I}\sin\left(\frac{\beta \pi }{2}\right)}{k_{D}\sin\left(\frac{\alpha \pi }{2}\right)}\right)}^{\frac{2}{\alpha +\beta }} & -k_{D}^{\frac{\beta }{\alpha +\beta }}\cos\left(\frac{\alpha \pi }{2}\right){\left(\frac{k_{I}\sin\left(\frac{\beta \pi }{2}\right)}{\sin\left(\frac{\alpha \pi }{2}\right)}\right)}^{\frac{\alpha }{\alpha +\beta }}\\ & -k_{I}^{\frac{\alpha }{\alpha +\beta }}\cos\left(\frac{\beta \pi }{2}\right){\left(\frac{k_{D}\sin\left(\frac{\alpha \pi }{2}\right)}{\sin\left(\frac{\beta \pi }{2}\right)}\right)}^{\frac{\beta }{\alpha +\beta }} \end{array}$$

*Furthermore, if β=1 then (40) reduces to*

(41) $$\begin{array}{c} k_{P}^{★}=\frac{1}{\mu_{2}}{\left(\frac{k_{I}}{k_{D}\sin\;\left(\frac{\alpha \pi }{2}\right)}\right)}^{\;\frac{2}{\alpha +1}}-k_{D}^{\frac{1}{\alpha +1}}\cos\;\left(\frac{\alpha \pi }{2}\right){\left(\frac{k_{I}}{\sin\;\left(\frac{\alpha \pi }{2}\right)}\right)}^{\;\frac{\alpha }{\alpha +1}}. \end{array}$$

*Note that if α=β=1 (i.e., integer-order PID consensus), then (40) further reduces to $k_{P}^{★}=\frac{k_{I}}{\mu_{2}k_{D}}$, or alternatively, $k_{I}<k_{I}^{★}=\mu_{2}k_{P}k_{D}$ for consensus stability on an undirected graph where $\mu_{2}$ is the second smallest eigenvalue of L [15].*

**Proof.**  Both rational and irrational fractional derivative and integral orders are considered in this theorem. Let us first consider the case in which the fractional derivative and integral orders are rational. Specifically, let $\alpha =p_{\alpha }/q_{\alpha }$ and $\beta =p_{\beta }/q_{\beta }$, where $p_{\alpha },q_{\alpha },p_{\beta },q_{\beta }\in Z^{+}$. Using the augmented state vector defined above, where $n=\left(2+\beta \right)r=2r+m_{\beta }p_{\beta }$ and $r=m_{\alpha }q_{\alpha }=m_{\beta }q_{\beta }$ is the least common multiple of $q_{\alpha }$ and $q_{\beta }$, with $m_{\alpha },m_{\beta }\in Z^{+}$, the closed-loop system is given by $D^{1/r}x=\left(A_{c}⊗I_{m}\right)x$, where $A_{c}=I_{N}⊗A-L⊗BK$ and $K=[k_{I},\ldots ,k_{P},\ldots ,k_{D},\ldots ]$. The characteristic equation $|sI_{nN}-A_{c}|=0$, where $s=\lambda^{1/r}$, can be written as

(42) $$\begin{array}{c} |sI_{nN}-A_{c}|=s^{n}\prod_{j=2}^{N}\left(s^{n}+\left(k_{D}s^{(\alpha +\beta )r}+k_{P}s^{\beta r}+k_{I}\right)\mu_{j}\right)=0. \end{array}$$

The Laplacian matrix L has a simple zero eigenvalue. Consequently, $A_{c}$ has exactly *n* zero eigenvalues associated with the consensus subspace, all contained in a single Jordan block. Therefore, consensus is achieved if and only if all remaining eigenvalues satisfy $|\arg\left(s_{i}\right)|>\pi /\left(2r\right)$. Substituting $s=\omega e^{i\pi /(2r)}$ and $\mu_{j}=a_{j}\pm ib_{j}$ into (42), and multiplying both sides by $s^{(1-\beta )r}$, yields

(43) $$\begin{array}{cc} \; & \left(-k_{D}\omega^{r(1+\alpha )}\sin\left(\frac{\alpha \pi }{2}\right)+k_{I}\omega^{r(1-\beta )}\sin\left(\frac{\beta \pi }{2}\right)\right)\mu_{j}\\ \; & +i\left(-\omega^{3r}+\left(k_{D}\omega^{r(1+\alpha )}\cos\left(\frac{\alpha \pi }{2}\right)+k_{P}\omega^{r}+k_{I}\omega^{r(1-\beta )}\cos\left(\frac{\beta \pi }{2}\right)\right)\mu_{j}\right)=0 \end{array}$$

From the real part $\omega^{r(\alpha +\beta )}=k_{I}\sin\left(\frac{\beta \pi }{2}\right)/\left(k_{D}\sin\left(\frac{\alpha \pi }{2}\right)\right)$. Substituting this into the imaginary part yields

(44) $$\begin{array}{c} -{\left(\frac{k_{I}\sin\left(\frac{\beta \pi }{2}\right)}{k_{D}\sin\left(\frac{\alpha \pi }{2}\right)}\right)}^{\frac{2}{\alpha +\beta }}+k_{D}^{\frac{\beta }{\alpha +\beta }}\cos\left(\frac{\alpha \pi }{2}\right){\left(\frac{k_{I}\sin\left(\frac{\beta \pi }{2}\right)}{\sin\left(\frac{\alpha \pi }{2}\right)}\right)}^{\frac{\alpha }{\alpha +\beta }}\mu_{j}\\ +\left(k_{P}+k_{I}^{\frac{\alpha }{\alpha +\beta }}{\left(\frac{k_{D}\sin\left(\frac{\alpha \pi }{2}\right)}{\sin\left(\frac{\beta \pi }{2}\right)}\right)}^{\frac{\beta }{\alpha +\beta }}\cos\left(\frac{\beta \pi }{2}\right)\right)\mu_{j}=0 \end{array}$$

Solving for $k_{P}$ yields (40) as a lower bound.

In the case where α and β are irrational, they can be approximated arbitrarily closely by infinitely many rational numbers $p_{\alpha }/q_{\alpha }$ and $p_{\beta }/q_{\beta }$, respectively. By Dirichlet’s approximation theorem, there exist infinitely many such approximations satisfying $|\alpha -p_{\alpha }/q_{\alpha }|<1/q_{\alpha }^{2}$ and $|\beta -p_{\beta }/q_{\beta }|<1/q_{\beta }^{2}$. Hence, by continuity, the result in (40) also holds for irrational α and β.    □

### 3.3. Feedback of Relative Integral States and Position and Fractional Velocity Errors Using a Reference Model with a Directed Graph

Given a reference model of the form

(45) $$\begin{array}{cc} {\dot{X}}_{r} & =B\left(X_{r}\right)\Omega_{r}, \end{array}$$

(46) $$\begin{array}{cc} I{\dot{\Omega }}_{r} & ={\mathrm{ad}}_{\Omega_{r}}^{*}I\Omega_{r}+u_{r} \end{array}$$

a second fractional cooperative control protocol $u_{i}$ is designed as

(47) $$\begin{array}{cc} u_{i} & =H\left(X_{i},\Omega_{i},I_{i}\right){\dot{X}}_{i}\\ & -G\left(X_{i},I_{i}\right)\left(K_{I}D^{-\beta }\sum_{j∼i}\left(X_{i}-X_{j}-X_{ij}^{d}\right)+K_{P}\left(X_{i}-X_{i}^{d}-X_{r}\right)+K_{D}D^{\alpha }\left(X_{i}-X_{r}\right)\right)+u_{r} \end{array}$$

results in the system as

(48) $$\begin{array}{c} {\ddot{\tilde{X}}}_{i}=-\left(K_{I}D^{-\beta }\sum_{j∼i}\left({\tilde{X}}_{i}-{\tilde{X}}_{j}\right)+K_{P}\left({\tilde{X}}_{i}-X_{r}\right)+K_{D}D^{\alpha }\left({\tilde{X}}_{i}-X_{r}\right)\right) \end{array}$$

where ${\tilde{X}}_{i}=X_{i}-X_{i}^{d}$.

**Theorem** **2.** *Assume N bodies communicate with a directed communication topology G(V,E,A) with Laplacian eigenvalues $\mu_{j}=a_{j}\pm ib_{j}$ and assume that $k_{I}$, $k_{P}$, and $k_{D}$ represent the gains for either the attitude or translational dynamics. Given a reference model of the form given in (45) and (46), and fractional derivative and integral orders α and β, the closed-loop system results in Mittag–Leffler consensus stability, if the communication graph G(V,E,A) contains a spanning tree, $k_{I}>0$, $k_{D}>0$, and $k_{P}>k_{P}^{★}$, where*

(49) $$\begin{array}{cc} k_{P}^{★} & =\max_{2\leq j\leq N}\{{\left(\frac{k_{I}A_{j}}{k_{D}\;\sin\;\left(\frac{\alpha \pi }{2}\right)}\right)}^{\;\frac{2}{\alpha +\beta }}\\ & -k_{D}^{\frac{\beta }{\alpha +\beta }}\cos\;\left(\frac{\alpha \pi }{2}\right){\left(\frac{k_{I}A_{j}}{\sin\;\left(\frac{\alpha \pi }{2}\right)}\right)}^{\;\frac{\alpha }{\alpha +\beta }}+k_{I}^{\frac{\alpha }{\alpha +\beta }}B_{j}{\left(\frac{k_{D}\;\sin\;\left(\frac{\alpha \pi }{2}\right)}{A_{j}}\right)}^{\;\frac{\beta }{\alpha +\beta }}\} \end{array}$$

*while*

(50) $$\begin{array}{c} A_{j}=a_{j}\sin\;\left(\frac{\beta \pi }{2}\right)+b_{j}\cos\;\left(\frac{\beta \pi }{2}\right),\;B_{j}=b_{j}\sin\;\left(\frac{\beta \pi }{2}\right)-a_{j}\cos\;\left(\frac{\beta \pi }{2}\right), \end{array}$$

*Note that the maximum over all non-zero Laplacian eigenvalues represents the intersection of N−1 hypersurfaces that yields the stability bound. In addition, for an undirected graph with real $\mu_{j}=a_{j}$, (49) reduces to*

(51) $$\begin{array}{c} k_{P}^{★}=\max_{2\leq j\leq N}\left\{\begin{array}{c} {\left(\frac{k_{I}\mu_{j}\sin\left(\frac{\beta \pi }{2}\right)}{k_{D}\sin\left(\frac{\alpha \pi }{2}\right)}\right)}^{\frac{2}{\alpha +\beta }}-k_{D}^{\frac{\beta }{\alpha +\beta }}\cos\left(\frac{\alpha \pi }{2}\right){\left(\frac{k_{I}\mu_{j}\sin\left(\frac{\beta \pi }{2}\right)}{\sin\left(\frac{\alpha \pi }{2}\right)}\right)}^{\frac{\alpha }{\alpha +\beta }}\\ -{\left(\mu_{j}k_{I}\right)}^{\frac{\alpha }{\alpha +\beta }}\cos\left(\frac{\beta \pi }{2}\right){\left(\frac{k_{D}\sin\left(\frac{\alpha \pi }{2}\right)}{\sin\left(\frac{\beta \pi }{2}\right)}\right)}^{\frac{\beta }{\alpha +\beta }} \end{array}\right\} \end{array}$$

*Furthermore, if β=1 (i.e., ${\mathrm{PID}}^{\alpha }$ consensus) then (49) reduces to*

(52) $$\begin{array}{cc} k_{P}^{★} & =\max_{2\leq j\leq N}\{{\left(\frac{k_{I}a_{j}}{k_{D}\sin\;\left(\frac{\alpha \pi }{2}\right)}\right)}^{\;\frac{2}{\alpha +1}}\\ \; & \;-k_{D}^{\frac{1}{\alpha +1}}\cos\;\left(\frac{\alpha \pi }{2}\right){\left(\frac{k_{I}a_{j}}{\sin\;\left(\frac{\alpha \pi }{2}\right)}\right)}^{\;\frac{\alpha }{\alpha +1}}+k_{I}^{\frac{\alpha }{\alpha +1}}\;b_{j}{\left(\frac{k_{D}\sin\;\left(\frac{\alpha \pi }{2}\right)}{a_{j}}\right)}^{\;\frac{1}{\alpha +1}}\}. \end{array}$$

*Also, if α=β=1 (i.e., integer-order PID consensus), then (49) further reduces to*

(53) $$k_{P}^{★}=\max_{2\leq j\leq N}\left\{\frac{k_{I}a_{j}}{k_{D}}+b_{j}\sqrt{\frac{k_{D}k_{I}}{a_{j}}}\right\}$$

*Thus, the condition that $k_{I}>0$, $k_{D}>0$, and $k_{P}>k_{P}^{★}$ in (53) is equivalent to $k_{P}>0$, $k_{D}>0$, and $k_{I}<k_{I}^{★}$ where*

(54) $$k_{I}^{★}=\min_{2\leq j\leq N}\left\{\frac{k_{D}}{a_{j}}\left(k_{P}+\frac{1}{2}{\left(\frac{k_{D}b_{j}}{a_{j}}\right)}^{2}-\frac{k_{D}b_{j}}{a_{j}}\sqrt{k_{P}+{\left(\frac{k_{D}b_{j}}{2a_{j}}\right)}^{2}}\right)\right\}$$

**Proof.**  Both rational and irrational fractional derivative and integral orders are considered in this theorem. For the rational-order case, let $\alpha =p_{\alpha }/q_{\alpha }$ and $\beta =p_{\beta }/q_{\beta }$, where $p_{\alpha },q_{\alpha },p_{\beta },q_{\beta }\in Z^{+}$. Using the augmented state vector defined above, where $n=\left(2+\beta \right)r=2r+m_{\beta }p_{\beta }$ and $r=m_{\alpha }q_{\alpha }=m_{\beta }q_{\beta }$ is the least common multiple of $q_{\alpha }$ and $q_{\beta }$, with $m_{\alpha },m_{\beta }\in Z^{+}$, the closed-loop system is given by $D^{1/r}x=\left(A_{c}⊗I_{m}\right)x$, where $A_{c}=I_{N}⊗\left(A-BK_{2}\right)-L⊗BK_{1}$, $K_{1}=\left[k_{I},0,\ldots \right]$, and $K_{2}=\left[0,\ldots ,k_{P},\ldots ,k_{D},\ldots \right]$. The characteristic equation $|sI_{nN}-A_{c}|=0$, where $s=\lambda^{1/r}$, can be written as $|sI_{nN}-A_{c}|=P\left(s\right)\prod_{j=2}^{N}\left(P\left(s\right)+k_{I}\mu_{j}\right)=0$, where $P\left(s\right)=s^{n}+k_{D}s^{(\alpha +\beta )r}+k_{P}s^{\beta r}$. This factorization follows from the modal decomposition of L and requires the nonzero eigenvalues to be semisimple, so that no nontrivial Jordan blocks occur in the disagreement dynamics. Therefore, the matrix $A_{c}$ has nN eigenvalues. $A_{c}$ has exactly βr zero eigenvalues contained in a single Jordan block. Therefore, it is necessary and sufficient for consensus that the remaining eigenvalues satisfy $|\mathrm{Arg}\left(s_{i}\right)|>\frac{\pi }{2r}$. Setting $s=\omega e^{i\pi /2r}$ and $\mu_{j}=a_{j}\pm ib_{j}$ in $s^{n}+k_{D}s^{(\alpha +\beta )r}+k_{P}s^{\beta r}+k_{I}\mu_{j}=0$ after multiplication by $s^{(1-\beta )r}$ yields

(55) $$\begin{array}{cc} \; & \left(-k_{D}\omega^{r(1+\alpha )}\sin\left(\frac{\alpha \pi }{2}\right)+k_{I}\omega^{r(1-\beta )}A_{j}\right)\\ & +i\left(-\omega^{3r}+k_{D}\omega^{r(1+\alpha )}\cos\left(\frac{\alpha \pi }{2}\right)+k_{P}\omega^{r}-k_{I}\omega^{r(1-\beta )}B_{j}\right)=0 \end{array}$$

From the real part $\omega^{r(\alpha +\beta )}=k_{I}\left(a_{j}\sin\left(\frac{\beta \pi }{2}\right)+b_{j}\cos\left(\frac{\beta \pi }{2}\right)\right)/\left(k_{D}\sin\left(\frac{\alpha \pi }{2}\right)\right)$. Substituting this into the imaginary part of (55) yields

(56) $$\begin{array}{c} k_{P}-{\left(\frac{k_{I}A_{j}}{k_{D}\sin\left(\frac{\alpha \pi }{2}\right)}\right)}^{\frac{2}{\alpha +\beta }}+k_{D}^{\frac{\beta }{\alpha +\beta }}\cos\left(\frac{\alpha \pi }{2}\right){\left(\frac{k_{I}A_{j}}{\sin\left(\frac{\alpha \pi }{2}\right)}\right)}^{\frac{\alpha }{\alpha +\beta }}-k_{I}^{\frac{\alpha }{\alpha +\beta }}B_{j}{\left(\frac{k_{D}\sin\left(\frac{\alpha \pi }{2}\right)}{A_{j}}\right)}^{\frac{\beta }{\alpha +\beta }}=0 \end{array}$$

where $A_{j}$ and $B_{j}$ are defined in (50). Solving for $k_{P}$ yields (49) as a lower bound.

The extension to irrational α and/or β follows from the same rational-approximation and continuity argument used in the proof of Theorem 1.    □

## 4. Collision Avoidance and Cohesion

### 4.1. Collision Avoidance

To prevent collisions among rigid bodies, a collision avoidance strategy must be incorporated into the consensus control protocols. First, a definition of a relative distance is required. The principal logarithm is used to define the relative configuration of the *j*th body with respect to the *i*th body. The relative configuration, expressed in exponential coordinates, is given by

(57) $$\begin{array}{c} {\left(X_{ij}\right)}^{\vee }=\log\;\left(g_{i}^{-1}g_{j}\right)\in \mathrm{se}\left(3\right). \end{array}$$

Using this relative configuration, the following relative distance metric is defined:

(58) $$\begin{array}{c} d_{ij}=\sqrt{X_{ij}^{T}WX_{ij}}, \end{array}$$

where $W=diag(W_{1},W_{2})$ is a 6×6 diagonal matrix, where $W_{1}=0_{3\times 3}$ and $W_{2}>0$ is a constant positive-definite matrix. This choice ensures that collision avoidance depends only on translational separation. The collision avoidance strategy employs two concentric safety regions around each agent. The outer region is defined by the collision detection radius (or sensing radius) $R_{s}$, while the inner region is defined by the collision radius $R_{c}$, where $R_{c}<R_{s}$. When the relative distance between two agents *i* and *j* satisfies $d_{ij}>R_{s}$, the collision avoidance controller remains inactive between agents *i* and *j* and only the formation controller is applied. Once the inter-agent distance enters the sensing region, i.e., $R_{c}<d_{ij}\leq R_{s}$, the collision avoidance controller is activated to generate a repulsive action that smoothly increases the separation between agents *i* and *j* only while preserving the desired formation objective. The collision radius $R_{c}$ represents the minimum allowable separation between two agents. Therefore, if $d_{ij}\leq R_{c}$, the safety constraint is violated and a collision is considered to have occurred. This two-layer safety mechanism enables early detection of potential collisions while maintaining a prescribed minimum separation between neighboring agents. In this section, the decentralized collision avoidance approach of [24] is adapted to the full-dynamics pose consensus problem using a positive-definite artificial potential function applied only to the translational dynamics.

Let $N_{i}^{\mathrm{col}}\subseteq \left\{1,\ldots ,N\right\}\setminus i$ denote the set of rigid bodies located within the sensing radius $R_{s}$ of the *i*th rigid body. The artificial Lennard–Jones potential associated with the *i*th body is defined as

(59) $$\begin{array}{c} U_{i}^{\mathrm{col}}=\sum_{j\in N_{i}^{\mathrm{col}}}U_{ij}^{\mathrm{col}}, \end{array}$$

where

(60) $$\begin{array}{c} U_{ij}^{\mathrm{col}}=\epsilon_{ij}\left[1+{\left(\frac{x_{ij}^{d}}{d_{ij}}\right)}^{12}-2{\left(\frac{x_{ij}^{d}}{d_{ij}}\right)}^{6}\right]. \end{array}$$

Here, $\epsilon_{ij}>0$ is a design parameter and $x_{ij}^{d}$ denotes the desired relative distance between the *i*th and *j*th bodies in the final formation. The collision avoidance force generated on the *i*th rigid body due to the proximity of the *j*th rigid body is given by

(61) $$\begin{array}{c} \phi_{ij}=-\left(\frac{dU_{ij}^{\mathrm{col}}}{dd_{ij}}\right)R_{i}^{T}\frac{r_{i}-r_{j}}{∥r_{i}-r_{j}∥}, \end{array}$$

where

(62) $$\begin{array}{c} \frac{dU_{ij}^{\mathrm{col}}}{dd_{ij}}=-\frac{12\epsilon_{ij}}{d_{ij}}\left[{\left(\frac{x_{ij}^{d}}{d_{ij}}\right)}^{12}-{\left(\frac{x_{ij}^{d}}{d_{ij}}\right)}^{6}\right]. \end{array}$$

In (61), $R_{i}\in \mathrm{SO}\left(3\right)$ is the rotation matrix from the body-fixed frame of the *i*th rigid body to the inertial frame, while $r_{i}$ and $r_{j}$ denote the inertial position vectors of the centers of mass of the *i*th and *j*th rigid bodies, respectively. The force in (61) vanishes when the inter-agent distance $d_{ij}$ equals the desired separation distance $x_{ij}^{d}$. Although, in theory, agents may become trapped in local minima of the artificial potential field, this phenomenon is rarely observed in practice due to measurement noise and other perturbations. The total collision avoidance force acting on the *i*th rigid body is therefore

(63) $$\begin{array}{c} \phi_{i}=\sum_{j\in N_{i}^{\mathrm{col}}}\phi_{ij}. \end{array}$$

### 4.2. Cohesion

To maintain group connectivity, a cohesion strategy is incorporated into the consensus controller [25]. Similar to the collision avoidance scheme, the cohesion inputs are generated using an artificial potential function. The cohesion forces are applied only to the translational dynamics. The cohesion strategy likewise employs two concentric regions to preserve the connectivity of the multi-agent system. The inner region is defined by the cohesion threshold radius $R_{\mathrm{coh}}$, while the outer region is defined by the maximum sensing (or communication) radius $R_{\max}$, where $R_{\mathrm{coh}}<R_{\max}$. When the relative distance between two neighboring agents satisfies $d_{ij}\leq R_{\mathrm{coh}}$, the cohesion controller remains inactive between agents *i* and *j* since the agents are already sufficiently close to maintain the desired formation. As the inter-agent distance enters the region $R_{\mathrm{coh}}<d_{ij}\leq R_{\max}$, the cohesion controller is activated to generate an attractive action that gradually draws agents *i* and *j* only closer together, thereby preventing excessive separation and preserving network connectivity. The maximum sensing radius $R_{\max}$ represents the largest allowable separation for maintaining communication or sensing between neighboring agents. Consequently, if $d_{ij}>R_{\max}$, the communication link is considered to be lost, and the desired network connectivity can no longer be guaranteed. This two-layer cohesion mechanism complements the collision avoidance strategy by preventing neighboring agents from becoming excessively separated while maintaining the connectivity required for coordinated motion.

Let $N_{i}^{\mathrm{coh}}\subseteq \left\{1,\ldots ,N\right\}\setminus i$ denote the set of rigid bodies located within the cohesion threshold radius $R_{\mathrm{coh}}$ of the *i*th rigid body, where *N* is the total number of rigid bodies in the formation. The artificial cohesion potential associated with the *i*th rigid body is defined as

(64) $$\begin{array}{c} U_{i}^{\mathrm{coh}}=\sum_{j\in N_{i}^{\mathrm{coh}}}U_{ij}^{\mathrm{coh}}, \end{array}$$

where

(65) $$\begin{array}{c} U_{ij}^{\mathrm{coh}}=\frac{k_{c}}{2}{\left(d_{ij}-x_{ij}^{d}\right)}^{2},\;d_{ij}=d\left(g_{i},g_{j}\right). \end{array}$$

Here, $k_{c}>0$ is a design parameter and $x_{ij}^{d}$ denotes the desired relative distance between the *i*th and *j*th rigid bodies in the final formation. The cohesion force generated on the *i*th rigid body due to the presence of the *j*th rigid body is given by

(66) $$\begin{array}{c} \psi_{ij}=-\left(\frac{dU_{ij}^{\mathrm{coh}}}{dd_{ij}}\right)R_{i}^{T}\frac{r_{i}-r_{j}}{∥r_{i}-r_{j}∥}, \end{array}$$

where

(67) $$\begin{array}{c} \frac{dU_{ij}^{\mathrm{coh}}}{dd_{ij}}=k_{c}\left(d_{ij}-x_{ij}^{d}\right). \end{array}$$

The force in (66) vanishes when the inter-agent distance $d_{ij}$ equals the desired separation distance $x_{ij}^{d}$. When $d_{ij}>x_{ij}^{d}$, the interaction produces an attractive effect that draws neighboring rigid bodies closer together. The total cohesion force acting on the *i*th rigid body is therefore

(68) $$\begin{array}{c} \psi_{i}=\sum_{j\in N_{i}^{\mathrm{coh}}}\psi_{ij}. \end{array}$$

Finally, the control law is modified to incorporate the collision avoidance and cohesion terms as

(69) $$\begin{array}{c} u_{i}^{\prime }=u_{i}+\left[\begin{array}{c} 0\\ \phi_{i}+\psi_{i} \end{array}\right], \end{array}$$

where $u_{i}^{\prime }$ denotes the control input of the *i*th rigid body when the collision avoidance and cohesion schemes are included.

## 5. Examples

In this section, the previously developed fractional-order ${\mathrm{PI}}^{\beta }D^{\alpha }$ cooperative control algorithms and their corresponding stability bounds are investigated. For all numerical simulations, the fde12 solver, which implements a predictor–corrector PECE method for fractional differential equations, is employed [26].

### 5.1. Example 1: Fractional ${\mathrm{PI}}^{\beta }D^{\alpha }$ Cooperative Pose Control with an Undirected Communication Graph

**Figure 2:**
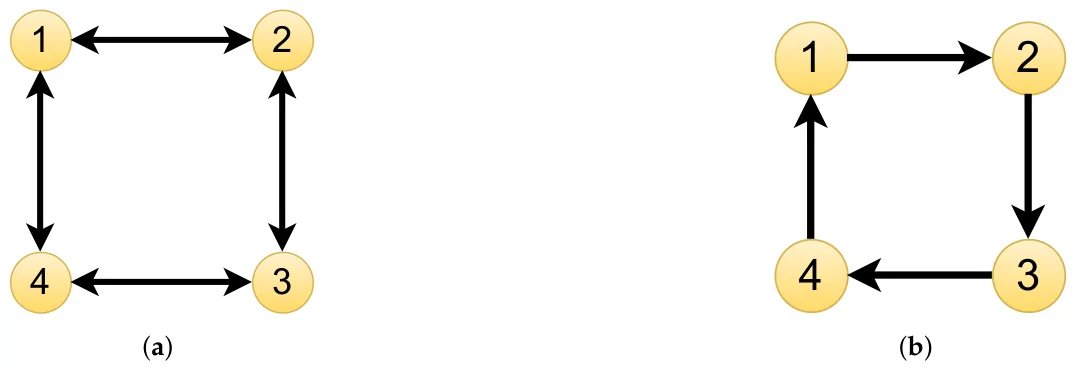
(**a**) An undirected cycle graph with four vertices; (**b**) a directed cycle graph with four vertices.

In this example, it is desired to study the control protocol (36), where the goal is for rigid bodies to align their attitudes, and the desired formation is selected such that the four rigid bodies achieve a tetrahedron-shaped formation with 40m edge length. To this end, consider an undirected ring graph topology of N=4 heterogeneous rigid bodies as depicted in Figure 2a with masses of $m_{1}=60$ kg, $m_{2}=26.8$ kg, $m_{3}=67.2$ kg and $m_{4}=50.4$ kg and inertia tensors of $J_{1}=\mathrm{diag}\left(16.25,10.00,16.25\right)$$\mathrm{kg}\cdot m^{2}$, $J_{2}=\mathrm{diag}\left(4.32,2.53,4.65\right)$$\mathrm{kg}\cdot m^{2}$, $J_{3}=\mathrm{diag}\left(16.57,19.04,13.66\right)$$\mathrm{kg}\cdot m^{2}$ and $J_{4}=\mathrm{diag}\left(8.10,15.49,11.50\right)$$\mathrm{kg}\cdot m^{2}$. The fractional derivative and integral orders in (36) are chosen as $\alpha =\beta =\frac{1}{2},$ corresponding to $p_{\alpha }=p_{\beta }=1$ and $q_{\alpha }=q_{\beta }=2$. Initial configurations in terms of exponential coordinates are selected as:

$$\begin{array}{cc} X_{01} & ={\left[-0.914,1.583,1.583,5.346,-9.260,6.574\right]}^{T},\\ X_{02} & ={\left[-0.914,-1.583,-1.583,-19.722,20.693,20.693\right]}^{T},\\ X_{03} & ={\left[-1.263,2.298,0.583,-14.987,-13.305,36.039\right]}^{T},\\ X_{04} & ={\left[0,-0.421,-0.412,21.479,-5.730,-4.365\right]}^{T}. \end{array}$$

with the initial velocity vectors set to zero. Define the augmented state vector for the *i*th agent as $z_{i}={\left[\begin{array}{cccc} \xi_{i1}^{T} & \xi_{i2}^{T} & \ldots & \xi_{i5}^{T} \end{array}\right]}^{T}$, where each $\xi_{ij}\in R^{6}$ denotes an augmented state block. The closed-loop system can be written as (39) where ζ=1/2, $K_{A}=\left[k_{I_{A}}\;k_{P_{A}}\;k_{D_{A}}\;0\;0\right]$, and $K_{T}=\left[k_{I_{T}}\;k_{P_{T}}\;k_{D_{T}}\;0\;0\right]$ while

$$\begin{array}{c} A=\left[\begin{array}{ccccc} 0 & 1 & 0 & 0 & 0\\ 0 & 0 & 1 & 0 & 0\\ 0 & 0 & 0 & 1 & 0\\ 0 & 0 & 0 & 0 & 1\\ 0 & 0 & 0 & 0 & 0 \end{array}\right],\;\;\;B=\left[\begin{array}{c} 0\\ 0\\ 0\\ 0\\ 1 \end{array}\right],\;\;\;L=\left[\begin{array}{cccc} 2 & -1 & 0 & -1\\ -1 & 2 & -1 & 0\\ 0 & -1 & 2 & -1\\ -1 & 0 & -1 & 2 \end{array}\right]. \end{array}$$

It is assumed the bodies have access to the relative pose configurations (relative attitudes and positions) and relative (angular and translational) velocities associated with its neighbors in the communication graph. Consequently, the measurable physical exponential coordinates correspond to the second and fourth augmented state blocks. To estimate the fractional states required for implementation of the proposed fractional-order cooperative controller, a fractional-order Luenberger-type observer is designed for each rigid body. The first augmented state block represents the fractional integral state generated through the Riemann–Liouville realization, as shown in Section 2.3, and is therefore not directly measurable. Consequently, the observer is designed only for the fractional velocity used in the feedback protocol. Define the reduced state vector as $z_{ri}={\left[\begin{array}{cccc} \xi_{i2}^{T} & \xi_{i3}^{T} & \xi_{i4}^{T} & \xi_{i5}^{T} \end{array}\right]}^{T}$. A fractional-order Luenberger observer is therefore designed as

(70) $$D_{t}^{0.5}{\hat{z}}_{ri}=\left(A_{r}⊗I_{6}\right){\hat{z}}_{ri}+\left(B_{r}⊗I_{6}\right)u_{i}+\left(L_{r}⊗I_{6}\right)\left(y_{i}-\left(C_{r}⊗I_{6}\right){\hat{z}}_{ri}\right),$$

where

$$A_{r}=\left[\begin{array}{cccc} 0 & 1 & 0 & 0\\ 0 & 0 & 1 & 0\\ 0 & 0 & 0 & 1\\ 0 & 0 & 0 & 0 \end{array}\right],\;B_{r}=\left[\begin{array}{c} 0\\ 0\\ 0\\ 1 \end{array}\right].$$

The output equation for the reduced subsystem is $y_{i}=\left(C_{r}⊗I_{6}\right)z_{ri}$, where

$$C_{r}=\left[\begin{array}{cccc} 1 & 0 & 0 & 0\\ 0 & 0 & 1 & 0 \end{array}\right].$$

The corresponding observability matrix is $O_{r}={\left[\begin{array}{cccc} C_{r}^{T} & {\left(C_{r}A_{r}\right)}^{T} & {\left(C_{r}A_{r}^{2}\right)}^{T} & {\left(C_{r}A_{r}^{3}\right)}^{T} \end{array}\right]}^{T}$ which satisfies $\mathrm{rank}(O_{r})=4$, implying that the complete reduced subsystem is observable. The observer gain matrix

$$L_{r}=\left[\begin{array}{cc} 1.8826 & -1.3632\\ -0.0581 & -1.0450\\ 0.5172 & 1.1780\\ 1.6394 & -2.5200 \end{array}\right]$$

is selected such that the eigenvalues of $A_{r}-L_{r}C_{r}$ satisfy Matignon’s stability condition, $\left|\arg\left(\lambda_{i}\left(A_{r}-L_{r}C_{r}\right)\right)\right|>\zeta \pi /2$. For ζ=0.5, this condition reduces to $\left|\arg\left(\lambda_{i}\left(A_{r}-L_{r}C_{r}\right)\right)\right|>\pi /4$. Finally, the observer gain for the complete six-degree-of-freedom system is $L_{\mathrm{obs}}=L_{r}⊗I_{6}$.

**Figure 3:**
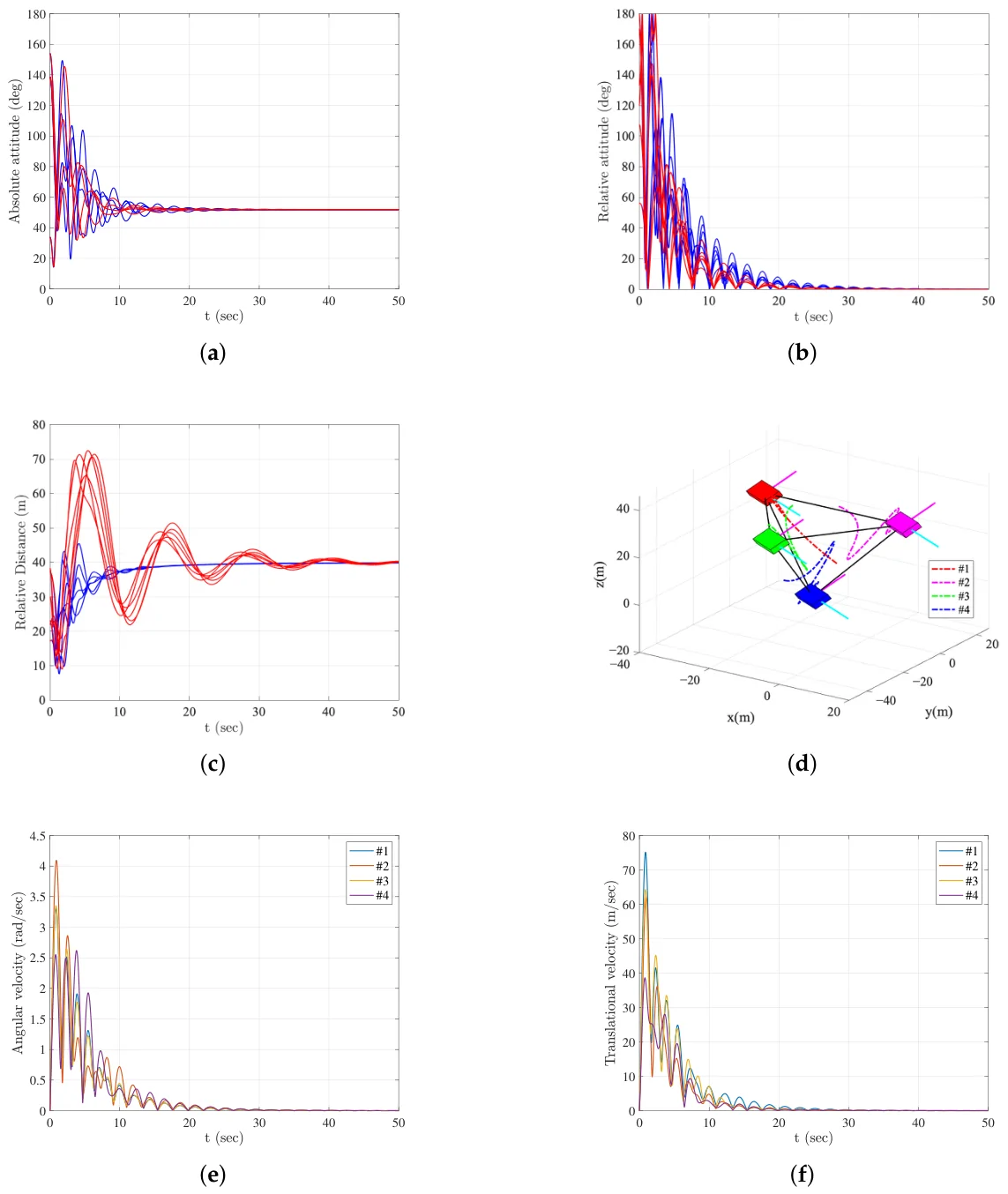
Example 1: Fractional (blue) vs. integer-order (red) PID pose cooperative control: PRAs of (**a**) absolute and (**b**) relative attitudes, (**c**) distances between each pair of bodies, (**d**) 3D trajectories and final orientations of the four bodies, and norms of (**e**) angular and (**f**) translational velocities of the bodies.

**Figure 4:**
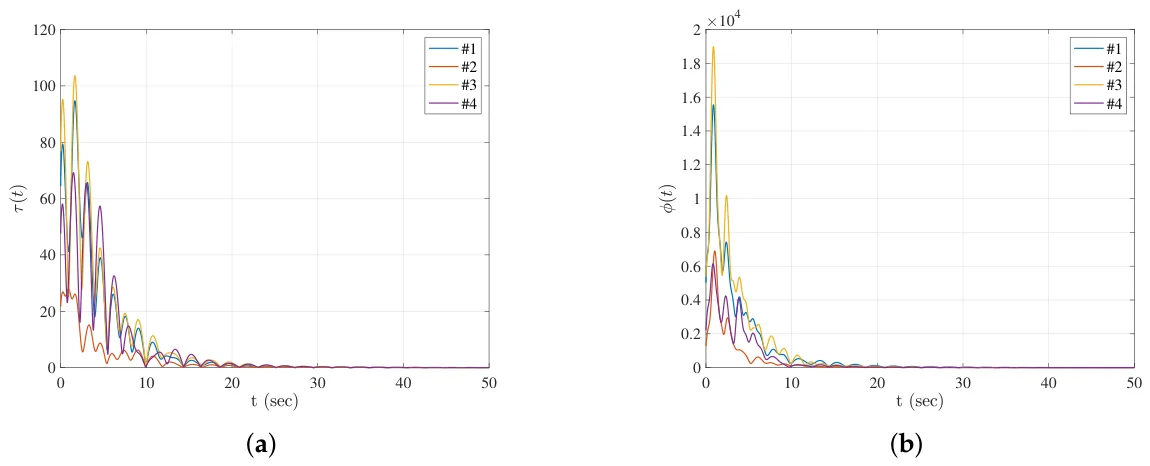
Example 1: Norms of the control inputs: (**a**) control torque and (**b**) control force.

The control gains in (36) are chosen as $k_{P_{A}}=0.6$, $k_{I_{A}}=0.2$, and $k_{D_{A}}=0.4$ for the attitude control, and $k_{P_{T}}=0.4$, $k_{I_{T}}=0.4$, and $k_{D_{T}}=1.4$ for the translational control. These choices result in a stable closed-loop system, with $k_{P_{A}}^{★}=-0.2750$ for the attitude control and $k_{P_{T}}^{★}=-1.0175$ for the translational control. After implementing the control inputs (36), all rigid bodies converge to the same constant attitude, as shown in Figure 3a,b. Figure 3c shows that the distances between each pair of bodies reach the desired value of 40 m. Additionally, the trajectories and final orientations of the four bodies are depicted in Figure 3d. Moreover, as depicted in Figure 3e,f, the norms of the angular and translational velocities converge to zero, and the system comes to rest asymptotically since the initial velocities are set to zero. Note that in Figure 3a–c, the performance of the fractional-order ${\mathrm{PI}}^{\beta }D^{\alpha }$ controller is compared with that of the integer-order PID controller using the same control gains. Figure 3c, in particular, shows that the fractional-order ${\mathrm{PI}}^{\beta }D^{\alpha }$ controller achieves a shorter settling time and smaller overshoot. Figure 4a,b present the norms of the control torques and control forces, respectively.

**Figure 5:**
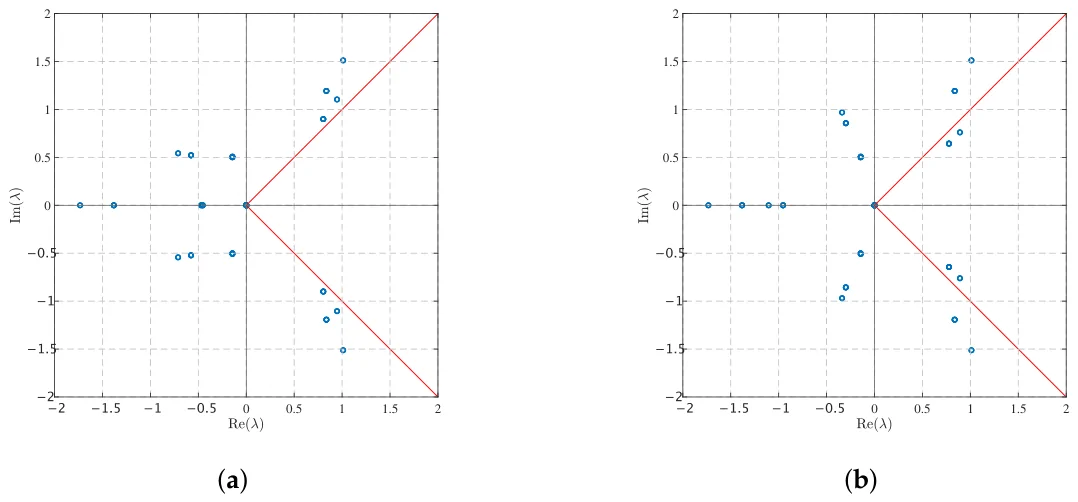
Example 1: Eigenvalues of the matrix *W* in (39) for the chosen gains in pose fractional ${\mathrm{PI}}^{\beta }D^{\alpha }$ cooperative control: (**a**) stable and (**b**) unstable. The red lines indicate the stability boundaries according to Matignon’s theorem, while the blue circles represent the eigenvalues.

The eigenvalues of matrix *W* in (39) for the chosen set of gains are shown in Figure 5a. Choosing another set of gains for the attitude feedback terms, $k_{P_{A}}=0.1$, $k_{I_{A}}=0.4$, and $k_{D_{A}}=0.1$, while keeping the translational gains unchanged, results in an unstable closed-loop system, with $k_{P_{A}}^{★}=7.71$, as shown in Figure 5b. Note that the stability criterion for the nonzero eigenvalues of *W* is $|\mathrm{Arg}\left(\lambda_{i}\right)|>\frac{\pi }{4}$, since the fractional order of the closed-loop system in (39) is $\zeta =\frac{1}{2}$.

**Figure 6:**
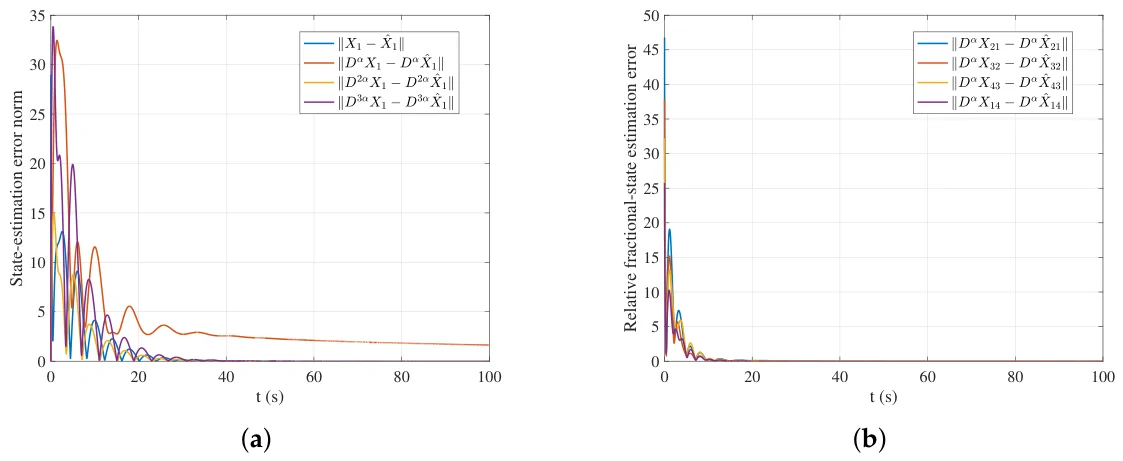
Estimation-error histories for the fractional-order Luenberger observer. (**a**) State-estimation errors for Rigid Body 1. (**b**) Relative fractional-state estimation errors for neighboring agent pairs, where $X_{ij}=X_{i}-X_{j}$ (e.g., $X_{21}=X_{2}-X_{1}$).

To further evaluate the performance of the fractional-order Luenberger observer, both the individual state-estimation errors and the relative fractional-state estimation errors are examined. Figure 6a shows the estimation errors for Rigid Body 1, where the state-estimation errors decay to zero, while the error associated with the fractional-derivative state decreases substantially over time but does not reach zero within the considered simulation interval. A similar error pattern is observed for the other bodies. Figure 6b shows that the relative fractional states converge to their corresponding estimated relative fractional states. These results indicate sufficient estimation accuracy for implementation of the derivative component of the proposed control law.

### 5.2. Example 2: Fractional ${\mathrm{PI}}^{\beta }D^{\alpha }$ Cooperative Pose Control with a Reference Trajectory Under a Directed Communication Graph

Now, consider a directed ring graph consisting of N=4 heterogeneous rigid bodies, as shown in Figure 2b, with the Laplacian matrix

(71) $$L=\left[\begin{array}{cccc} 1 & 0 & 0 & -1\\ -1 & 1 & 0 & 0\\ 0 & -1 & 1 & 0\\ 0 & 0 & -1 & 1 \end{array}\right],$$

which contains a spanning tree and has the spectrum spec(L)={0,1.0±1.0i,2.0}. The rigid bodies are assumed to have the same mass and inertia properties as those considered in the previous example. The control gains are chosen as $k_{P_{A}}=1.2$, $k_{I_{A}}=0.5$, and $k_{D_{A}}=1.2$ for the attitude controller, and $k_{P_{T}}=1.0$, $k_{I_{T}}=0.4$, and $k_{D_{T}}=1.2$ for the translational controller. These choices result in a stable closed-loop system, with $k_{P_{A}}^{★}=0.494$ for the attitude dynamics and $k_{P_{T}}^{★}=0.366$ for the translational dynamics. Additionally, the fractional derivative and integral orders are selected as α=0.5=1/2$\left(p_{\alpha }=1,;q_{\alpha }=2\right)$ and β=1$\left(p_{\beta }=1,;q_{\beta }=1\right)$, respectively.

To estimate the fractional states required by the proposed fractional-order cooperative controller, a fractional-order Luenberger-type observer is again employed for each agent. Define the augmented state vector as $z_{i}={\left[\begin{array}{cccc} \xi_{i1}^{T} & \xi_{i2}^{T} & \ldots & \xi_{i6}^{T} \end{array}\right]}^{T}$, where $\xi_{ij}\in R^{6}$ for j=1,…,6. The first two augmented states arise from the Riemann–Liouville realization of the fractional operators, as shown in Section 2.3. Therefore, the reduced state vector is defined as $z_{ri}={\left[\begin{array}{cccc} \xi_{i3}^{T} & \xi_{i4}^{T} & \xi_{i5}^{T} & \xi_{i6}^{T} \end{array}\right]}^{T}$, and the output equation is $y_{i}=\left(C_{r}⊗I_{6}\right)z_{ri}$, where

$$C_{r}=\left[\begin{array}{cccc} 1 & 0 & 0 & 0\\ 0 & 0 & 1 & 0 \end{array}\right].$$

The observability matrix associated with the scalar pair $\left(A_{r},C_{r}\right)$ satisfies $\mathrm{rank}(O_{r})=4$. Consequently, the observability matrix of the complete reduced subsystem is $O_{r}⊗I_{6}$, and $\mathrm{rank}(O_{r}⊗I_{6})=24$. Hence, the reduced subsystem is completely observable. The observer gain matrix

$$L_{r}=\left[\begin{array}{cc} 1.8859 & -1.3691\\ -0.0530 & -1.0801\\ 0.5091 & 1.3196\\ 1.6267 & -2.1797 \end{array}\right]$$

is selected such that the eigenvalues of $A_{r}-L_{r}C_{r}$ satisfy Matignon’s stability condition, $\left|\arg\left(\lambda_{i}\left(A_{r}-L_{r}C_{r}\right)\right)\right|>\pi /4$. Hence, the observer error is globally Mittag–Leffler stable.

#### Reference Trajectory and Its Fractional Derivatives

Consider the following spatial reference trajectory:

(72) $$X_{r}\left(t\right)={\left[\begin{array}{cccccc} 0 & 0 & 0 & v_{0}t & a_{1}\sin\left(\omega t\right) & a_{2}\cos\left(\omega t\right) \end{array}\right]}^{T},$$

where $v_{0}$ denotes the constant forward velocity, $a_{1}$ and $a_{2}$ are the amplitudes of the sinusoidal motions in the *y*- and *z*-directions, respectively, and ω denotes their common angular frequency. Using the fractional derivative properties

(73) $$D^{\alpha }t=\frac{t^{1-\alpha }}{\Gamma (2-\alpha )},$$

(74) $$D^{\alpha }\sin(\omega t)=\omega^{\alpha }\sin\left(\omega t+\frac{\alpha \pi }{2}\right),$$

(75) $$D^{\alpha }\cos\left(\omega t\right)=\omega^{\alpha }\cos\left(\omega t+\frac{\alpha \pi }{2}\right),$$

the fractional derivative of the reference trajectory is

(76) $$D^{\alpha }X_{r}\left(t\right)={\left[\begin{array}{cccccc} 0 & 0 & 0 & \frac{v_{0}t^{1-\alpha }}{\Gamma (2-\alpha )} & a_{1}\omega^{\alpha }\sin\left(\omega t+\frac{\alpha \pi }{2}\right) & a_{2}\omega^{\alpha }\cos\left(\omega t+\frac{\alpha \pi }{2}\right) \end{array}\right]}^{T}.$$

For α=0, $D^{0}X_{r}\left(t\right)=X_{r}\left(t\right)$ while for α=1,

(77) $$D^{1}X_{r}\left(t\right)={\left[\begin{array}{cccccc} 0 & 0 & 0 & v_{0} & a_{1}\omega \cos\left(\omega t\right) & -a_{2}\omega \sin\left(\omega t\right) \end{array}\right]}^{T},$$

which corresponds to the integer velocity vector. Therefore, the feedforward reference input is calculated as the second derivative of the desired trajectory,

(78) $$u_{r}\left(t\right)={\ddot{X}}_{r}\left(t\right)={\left[\begin{array}{cccccc} 0 & 0 & 0 & 0 & -a_{1}\omega^{2}\sin\left(\omega t\right) & -a_{2}\omega^{2}\cos\left(\omega t\right) \end{array}\right]}^{T}.$$

In this paper, the reference trajectory parameters are chosen as $v_{0}=1\;m/s$, $a_{1}=10\;m$, $a_{2}=5\;m$, and ω=0.2rad/s.

**Figure 7:**
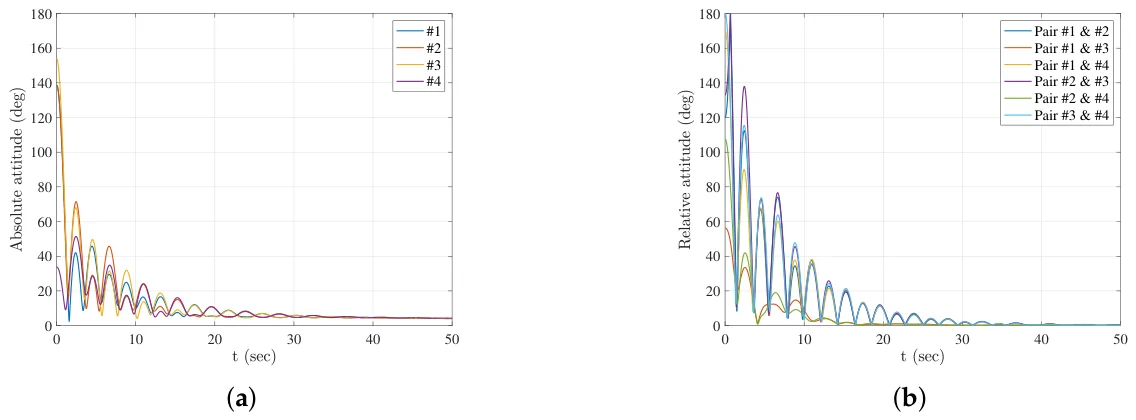

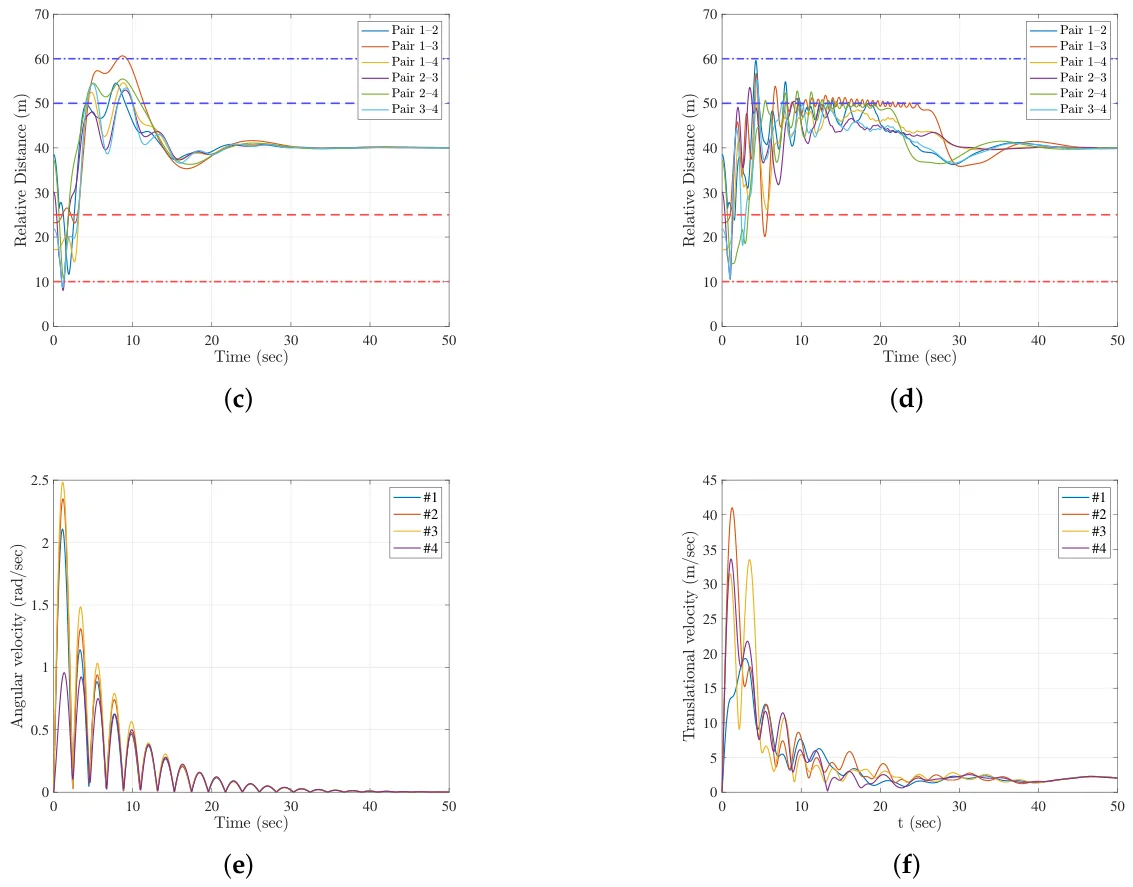
Example 2: Fractional ${\mathrm{PI}}^{\beta }D^{\alpha }$ pose cooperative control of a MARBS with a reference trajectory and directed graph: PRAs of (**a**) absolute and (**b**) relative attitudes; pairwise distances (**c**) without and (**d**) with collision avoidance and cohesion, with thresholds $R_{c}=10\;m$ (red dash-dotted), $R_{s}=25\;m$ (red dashed), $R_{\mathrm{coh}}=50\;m$ (blue dashed), and $R_{\max}=60\;m$ (blue dash-dotted); and norms of (**e**) angular and (**f**) translational velocities.

After implementing the control protocol (47), all rigid bodies converge to the same attitude, as shown in Figure 7a,b. The pairwise distances between the bodies converge to the desired value of 40m, as shown in Figure 7c without collision avoidance and cohesion, and in Figure 7d with collision avoidance (where $\epsilon_{ij}=0.2$ in (62)) and cohesion (where $k_{c}=4.5$ in (65)). Moreover, the norms of the angular velocities converge to zero, as depicted in Figure 7e, while the norms of the translational velocities converge to a nonzero consensus value, as shown in Figure 7f.

**Figure 8:**
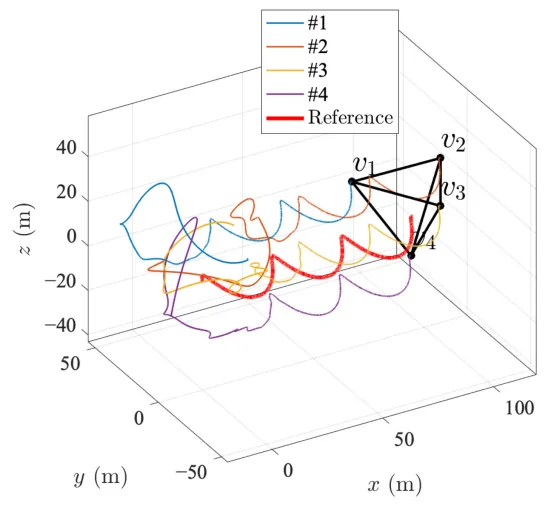
Example 2: Three-dimensional trajectories of the four rigid bodies under the proposed fractional ${\mathrm{PI}}^{\beta }D^{\alpha }$ cooperative controller (47). The red curve denotes the reference trajectory, while the remaining four curves represent the trajectories of the rigid bodies. The results demonstrate that all bodies successfully track the prescribed reference trajectory while maintaining the desired formation throughout the maneuver.

Figure 8 illustrates the three-dimensional trajectories of the four rigid bodies, together with the reference trajectory (red), for the case without collision avoidance and cohesion. It can be observed that all four bodies accurately track the desired trajectory while preserving the prescribed formation throughout the simulation, thereby demonstrating the effectiveness of the proposed fractional ${\mathrm{PI}}^{\beta }D^{\alpha }$ cooperative control strategy.

## 6. Conclusions

This paper addressed the cooperative pose control problem for multi-agent rigid-body systems using fractional-order ${\mathrm{PI}}^{\beta }D^{\alpha }$ controllers under both undirected and directed communication topologies. The rigid-body pose configurations were represented using exponential coordinates on the Lie group SE(3). Analytical stability bounds were derived using Matignon’s theorem in terms of the proportional, derivative, and integral gains for both attitude and translational control, together with the fractional orders, thereby guaranteeing global Mittag–Leffler stability. These results provide a systematic and theoretically framework for sequentially tuning the controller gains and fractional orders to achieve stable cooperative behavior. Since the fractional derivative states are not directly measurable, a Luenberger observer was incorporated to estimate these states from the measurable pose exponential coordinates, enabling practical implementation of the proposed feedback protocols. Simulation results demonstrated the effectiveness of the proposed approach for formation establishment while incorporating cohesion and collision-avoidance strategies based on artificial potential functions. Compared with conventional integer-order PID control, the proposed fractional-order controllers provide additional flexibility in shaping the closed-loop response and improving control performance.

## Data Availability

The original contributions presented in this study are included in the article. Further inquiries can be directed to the corresponding author.

## Appendix A. Time Derivative of the Matrix U(X)

In this section, the analytical expression for $\dot{U}\left(X\right)$ is derived. Consider the kinematics

(A1) $$\dot{X}=B\left(X\right)\Omega .$$

Define

(A2) $$C={\mathrm{ad}}_{X}=\left[\begin{array}{cc} \Theta^{\times } & 0_{3\times 3}\\ p^{\times } & \Theta^{\times } \end{array}\right],$$

so that

(A3) $$B\left(X\right)=I_{6}+\frac{1}{2}C+a\left(\theta \right)C^{2}+b\left(\theta \right)C^{4},$$

where θ=∥Θ∥, and

(A4) $$a(\theta )=\frac{1}{\theta^{2}}\left(2-\frac{3}{4}\theta \cot\frac{\theta }{2}-\frac{\theta^{2}}{8}\csc^{2}\frac{\theta }{2}\right),$$

(A5) $$b(\theta )=\frac{1}{\theta^{4}}\left(1-\frac{1}{4}\theta \cot\frac{\theta }{2}-\frac{\theta^{2}}{8}\csc^{2}\frac{\theta }{2}\right).$$

Since $C={\mathrm{ad}}_{X}$,

(A6) $$\dot{C}={\mathrm{ad}}_{\dot{X}}=\left[\begin{array}{cc} {\dot{\Theta }}^{\times } & 0_{3\times 3}\\ {\dot{p}}^{\times } & {\dot{\Theta }}^{\times } \end{array}\right]={\mathrm{ad}}_{B(X)\Omega }.$$

Moreover,

(A7) $$\dot{\theta }=\frac{\Theta^{T}\dot{\Theta }}{\theta },\;\theta \neq 0,$$

which gives

(A8) $$\dot{a}\left(\theta \right)=a^{\prime }\left(\theta \right)\dot{\theta },\;\dot{b}\left(\theta \right)=b^{\prime }\left(\theta \right)\dot{\theta }.$$

The derivatives of a(θ) and b(θ) are

(A9) $$a^{\prime }\left(\theta \right)=-\frac{4}{\theta^{3}}+\frac{3}{4\theta^{2}}\cot\frac{\theta }{2}+\frac{3}{8\theta }\csc^{2}\frac{\theta }{2}+\frac{1}{8}\csc^{2}\frac{\theta }{2}\cot\frac{\theta }{2},$$

(A10) $$b^{\prime }\left(\theta \right)=-\frac{4}{\theta^{5}}+\frac{3}{4\theta^{4}}\cot\frac{\theta }{2}+\frac{3}{8\theta^{3}}\csc^{2}\frac{\theta }{2}+\frac{1}{8\theta^{2}}\csc^{2}\frac{\theta }{2}\cot\frac{\theta }{2}.$$

Differentiating B(X) with respect to time yields

(A11) $$\dot{B}=\frac{1}{2}\dot{C}+\dot{a}\;C^{2}+a\frac{d(C^{2})}{dt}+\dot{b}\;C^{4}+b\frac{d(C^{4})}{dt}.$$

Using the product rule,

(A12) $$\frac{d(C^{2})}{dt}=\dot{C}\;C+C\;\dot{C},$$

(A13) $$\frac{d(C^{4})}{dt}=\dot{C}\;C^{3}+C\;\dot{C}\;C^{2}+C^{2}\dot{C}\;C+C^{3}\dot{C}.$$

Therefore,

(A14) $$\begin{array}{cc} \dot{B}= & \frac{1}{2}\dot{C}+a^{\prime }\left(\theta \right)\dot{\theta }\;C^{2}+a\left(\theta \right)\left(\dot{C}\;C+C\;\dot{C}\right)\\ & +b^{\prime }\left(\theta \right)\dot{\theta }\;C^{4}+b\left(\theta \right)\left(\dot{C}\;C^{3}+C\;\dot{C}\;C^{2}+C^{2}\dot{C}\;C+C^{3}\dot{C}\right). \end{array}$$

Substituting

(A15) $$\dot{\theta }=\frac{\Theta^{T}\dot{\Theta }}{\theta },$$

gives the final expression

(A16) $$\begin{array}{cc} \dot{B}\left(X\right)= & \frac{1}{2}\dot{C}+a^{\prime }\left(\theta \right)\frac{\Theta^{T}\dot{\Theta }}{\theta }C^{2}+a\left(\theta \right)\left(\dot{C}\;C+C\;\dot{C}\right)\\ \; & +b^{\prime }\left(\theta \right)\frac{\Theta^{T}\dot{\Theta }}{\theta }C^{4}+b\left(\theta \right)\left(\dot{C}\;C^{3}+C\;\dot{C}\;C^{2}+C^{2}\dot{C}\;C+C^{3}\dot{C}\right), \end{array}$$

where

(A17) $$C={\mathrm{ad}}_{X},\;\dot{C}={\mathrm{ad}}_{B(X)\Omega }.$$

Finally, letting $U=B^{-1}$, and differentiating both sides of $BU=I_{6}$ gives

(A18) $$\dot{B}\;U+B\;\dot{U}=0.$$

Hence,

(A19) $$B\dot{U}=-\dot{B}\;U,$$

and premultiplying by $B^{-1}=U$ yields

(A20) $$\dot{U}=-U\dot{B}\;U.$$
